# The efficacy and safety of percutaneous coronary intervention via distal transradial access in the treatment of coronary heart disease patients

**DOI:** 10.12669/pjms.41.9.12603

**Published:** 2025-09

**Authors:** Dongqin Ge, Gaojun Cai, Feng Li, Qiuwei Zhang

**Affiliations:** 1 Dongqin Ge Department of Interventional Catheterization Room, Wujin People’s Hospital, Changzhou, Jiangsu Province 213000, P.R. China; 2 Gaojun Cai Department of Cardiovascular, Wujin People’s Hospital, Changzhou, Jiangsu Province 213000, P.R. China; 3 Feng Li Department of Cardiovascular, Wujin People’s Hospital, Changzhou, Jiangsu Province 213000, P.R. China; 4 Qiuwei Zhang Department of Interventional Catheterization Room, Wujin People’s Hospital, Changzhou, Jiangsu Province 213000, P.R. China

**Keywords:** Coronary heart disease, Distal, Percutaneous coronary intervention, Transradial access

## Abstract

**Objective:**

To explore the efficacy and safety of percutaneous coronary intervention (PCI) through the distal transradial access (dTRA) for the treatment of coronary heart disease (CHD).

**Methodology:**

This retrospective cohort analysis included records of CHD patients who underwent PCI in Wujin People’s Hospital between January, 2023 to December, 2024. Patients (n=107) were grouped based on the access method. Patients (n=55) who received dTRA comprised the dTRA group, and patients (n=52) who received standard transradial access (TRA) comprised the TRA group. Basic clinical information, surgery-related indicators, and the incidence and types of complications were compared between the dTRA and TRA groups.

**Results:**

There was no statistically significant difference in the initial puncture success rate, puncture frequency, contrast agent dosage, X-ray exposure time, and surgical time between the two groups (all P>0.05). The dTRA group had a longer puncture time than the TRA group, but the postoperative compression time was significantly reduced (both P<0.05). There was no significant difference in the incidence of individual complications between the two groups (both P>0.05). However, the total number of complications in the dTRA group was significantly lower than that in the TRA group (10.9% vs. 28.8%) (P<0.05).

**Conclusions:**

Compared with TRA, dTRA has certain advantages in the PCI treatment of CHD, especially in terms of reducing the postoperative compression time and the overall incidence of complications.

## INTRODUCTION

Percutaneous coronary intervention (PCI) has become one of the leading methods for treating coronary heart disease (CHD), and can significantly improve the prognosis of patients.^1^,^2^ Importantly, choosing the appropriate vascular approach is the key to ensuring the success of PCI surgery.^3^,^4^ The classic transradial artery approach (TRA), one of the commonly used accesses for PCI surgery, has the advantages of a higher puncture success rate, convenient hemostasis, unrestricted postoperative activity, and fast recovery.^3^-^5^ However, this method is associated with certain complications such as arteriovenous fistula, local hematoma, and radial artery occlusion (RAO).^6^,^7^ Studies have showed that the incidence of RAO on the first day after TRA is as high as 17.4%,^4^ while the incidence of complications at the access site may reach 33.1%.^5^

Distal transradial access (dTRA) technology was first reported in 2014.^8^ This method was shown to have unique anatomical advantages. The blood vessels in the radial area are relatively shallow, making it easy to compress them to stop the bleeding. In addition, the hand has abundant collateral circulation, which can effectively reduce the risk of local complications after surgery, especially RAO, making dTRA a method of choice for coronary angiography and PCI.^8^,^9^

Although both approaches are used in clinical practice, there is currently limited direct comparative research on the effectiveness and safety of dTRA and TRA for PCI treatment of CHD.^8^-^10^ This study aimed to compare the safety and effectiveness of dTRA and TRA to further optimize the PCI treatment plan for CHD and improve treatment effectiveness. Although comparative studies on dTRA and TRA have been published globally, this study contributes one of the few retrospective analyses focusing on PCI via dTRA in Jiangsu Province, China, and includes predominantly middle-aged and elderly patients with NYHA class II–IV cardiac function. As such, it provides region-specific, confirmatory evidence of dTRA’s safety and feasibility in real-world clinical practice in non-tertiary hospitals.

## METHODOLOGY

This retrospective analysis reviewed records of CHD patients who underwent PCI surgery in Wujin People’s Hospital from January 2023 to December 2024. Patients were retrospectively divided into the dTRA and the TRA group according to different access methods. Only patients who successfully completed PCI via the assigned access route were included in the analysis. Cases involving access failure with crossover to an alternative route or aborted procedures were excluded.

### Ethical approval:

The ethics committee of Wujin People’s Hospital approved this retrospective study with the number: 2025-SR-170; Date: April 16, 2025.

### Inclusion Criteria:

- First PCI treatment.
- Age over 40 years.
- Individuals with heart function grades II-IV.
- Received dTRA or TRA.
- Complete clinical data.

### Exclusion Criteria:

- History of previous cardiovascular intervention therapy.
- Severe liver and kidney dysfunction.
- Blood system diseases.
- Change of access method midway and failure of PCI surgery.

### Procedure

The patient was positioned supine with the right upper limb extended 30 degrees. After routine disinfection, the left hand was placed on the right side of the groin with the back of the hand facing upwards. The right hand was fixed on a wooden board secured under the shoulder, and a towel was wrapped around the wrist. For patients undergoing TRA, the puncture was performed at the most prominent radial artery pulsation point 2-3cm above the wrist crease. For patients with dTRA, the puncture point was located at the anatomical snuffbox region of the distal radial artery, within the triangular area formed by the extensor hallucis longus tendon, extensor hallucis brevis tendon, and radial styloid process, where there is a significant radial artery pulsation. After subcutaneous injection of 1-3 ml of 2% lidocaine hydrochloride local anesthetic, the needle (Cordis, USA) was pointed towards the site of maximum pulse.

**Fig.1:**
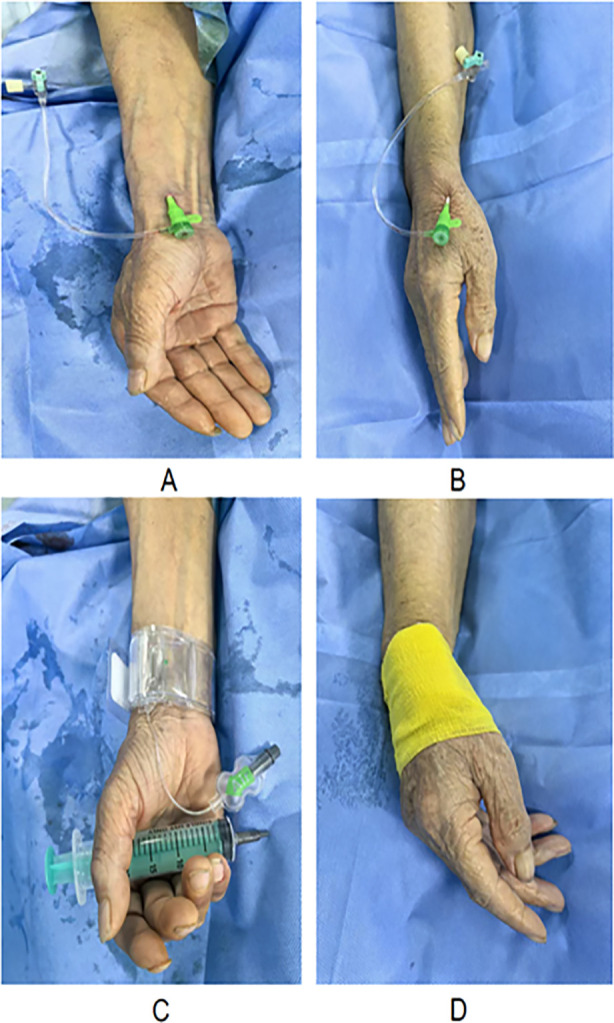
**A:** Transradial access; **B:** Distal transradial access; **C:** Transradial access artery compression; **D:** Distal transradial access artery compression.

After arterial puncture, a 0.018-inch soft metal wire was gently introduced while maintaining the patient’s wrist in a semi-abducted and extended position. The six French radial artery sheath was inserted into the posterior position (Fig.1A and Fig.1B). To prevent radial artery spasm and thrombosis, all patients received a combination therapy of weight-adjusted unfractionated heparin (40 to 70 U/kg, up to 5000) and 200 μg nitroglycerin. Subsequently, the coronary angiography catheter was delivered through a sheath to the opening of the coronary artery for coronary angiography to determine the location, severity, and course of the lesion. Based on the imaging results, the appropriate guiding catheters, wires, and stents were selected to complete the PCI surgical procedure. After the surgery, the sheath was removed.

For hemostasis, patients in the TRA group were treated using a radial artery compression device (Fig.1C), whereas those in the dTRA group had the puncture site covered with gauze and secured using three layers of Ulex bandages in a cross-compression fashion (Fig.1D). The compression time was adjusted according to the specific situation of the patient. Basic information including gender, age, previous medical history (such as hypertension, diabetes, blood lipids, etc.), smoking history and cardiac function classification were collected. All procedures were performed by two senior interventional cardiologists, each with more than five years of experience in transradial and distal transradial interventions, and over 200 dTRA cases completed prior to the study. No procedures were performed by trainees or junior staff.

### Collected indicators:

Surgery-related data included success rate of one puncture (defined as the proportion of successful sheath insertion in one puncture), puncture time (from local anesthesia to successful sheath insertion, time required for blood to be drawn back), number of puncture attempts (defined as the number of attempts required to successfully insert the sheath), contrast agent dose, X-ray exposure time, surgical time (from puncture to sheath removal at the end of surgery), postoperative compression time (defined as the duration from the initiation of the first compression maneuver immediately after sheath removal, until the complete removal of the compression device or bandage without signs of rebleeding; all patients achieved hemostasis with a single compression, and no repeated compression was required).

Postoperative complications included radial artery spasm (unable to reach radial artery pulsation after puncture), local hematoma (palpable mass around the radial artery puncture site), pseudoaneurysm, arteriovenous fistula, RAO (confirmed by ultrasound one week after surgery to have no forward blood flow in the radial artery).^9^,^10^

### Statistical analysis:

Categorical variables were displayed as frequency and percentage. Continuous variables with normal distribution were represented as mean ± SD, while continuous variables without normal distribution were represented as median with interquartile range. The Kolmogorov-Smirnov test was used to evaluate the normality of the data. Categorical variables were analyzed using the chi-square test or Fisher’s exact test, while continuous variables were analyzed using the independent *t*-test or the Mann-Whitney *U* test. Statistical analysis was conducted using SPSS (version 22.0, SPSS. Inc., Chicago, Illinois). Double tailed P<0.05 was considered statistically significant.

## RESULTS

This retrospective analysis included data from 107 eligible patients (63 males and 44 females) aged 47-84 years (average age of 66.8 ± 7.9 years). Of them, 46.7% had hypertension, 27.1% had diabetes, and 32.7% presented with abnormal lipid profiles (Table-I). In terms of heart function grading, 26 patients were classified as Grade-II, 60 as Grade-III, and 21 as Grade-IV. Fifty-five patients received dTRA and 52 patients received TRA, with no significant differences in gender, age, proportion of underlying diseases, proportion of smoking history, and cardiac function grading between the two groups (all P>0.05) (Table-I).

**Table-I:** Comparison of basic characteristics between two groups of patients.

Characteristics	dTRA group (n=55)	TRA group (n=52)	χ²/t	P
Male (Yes), n(%)	33 (60.0)	30 (57.7)	0.059	0.808
Age (years), mean±SD	65.9±8.5	67.8±7.3	-1.192	0.236
Hypertension (Yes), n(%)	28 (50.9)	22 (42.3)	0.794	0.373
Diabetes (Yes), n(%)	18 (32.7)	11 (21.2)	1.812	0.178
Abnormal blood lipids (yes), n(%)	20 (36.4)	15 (28.8)	0.686	0.407
Smoking history (yes), n(%)	27 (49.1)	24 (46.2)	0.092	0.761
Classification of cardiac function, n(%)			1.730	0.421
II	16 (29.1)	10 (19.2)		
III	30 (54.5)	30 (57.7)		
IV	9 (16.4)	12 (23.1)		

TRA: transradial access; dTRA: distal transradial access.

There were no significant differences in the initial puncture success rate, number of punctures, contrast agent dosage, X-ray exposure time, and surgical time between the dTRA and the TRA groups (all P>0.05) Table-II. However, the puncture time in the dTRA group was significantly higher than that in the TRA group (5 (4-5) vs. 3 (2-5) min). In contrast, the postoperative compression time was significantly lower than that in the TRA group (4 (3-5) vs. 6 (5-8) h) (both P<0.05).

**Table-II:** Comparison of surgical outcomes between two groups of patients.

Variables	dTRA group (n=55)	TRA group (n=52)	χ²/t/Z	P
Initial puncture successful	48 (87.3)	50 (92.6)	1.705	0.192^#^
Puncture time (min)	5 (4-5)	3 (2-5)	-4.304	<0.001
Number of puncture attempts	1 (1-1)	1 (1-1)	-1.671	0.095
Contrast agent dosage (ml)	53.5±11.9	55.0±14.2	-0.575	0.566
X-ray exposure time (min)	9 (8-12)	10.5 (8.5-13.5)	-0.596	0.551
Surgical time (min)	47.1±5.9	48.4±7.1	-1.038	0.302
Postoperative compression time (h)	4 (3-5)	6 (5-8)	-5.384	<0.001

# Fisher’s Exact Test. TRA: transradial access; dTRA: distal transradial access; Min: minute; h: hour.

dTRA was associated with a significantly lower rate of complications (six patients (10.9%) in the dTRA group vs 15 patients (28.8%) in the TRA group, P<0.05), Table-III. There was no considerable difference in the incidence of individual complications between the two groups (P>0.05).

**Table-III:** Comparison of complications between the two groups.

Complications	dTRA group (n=55)	TRA group (n=52)	χ²	P
Radial artery spasm	2 (3.6)	4 (7.7)	0.241	0.623^#^
Local hematoma	1 (1.8)	2 (3.8)	0.002	0.961^#^
Pseudoaneurysm	2 (3.6)	2 (3.8)	0.000	1.000^#^
Arteriovenous fistula	0 (0)	2 (3.8)	0.569	0.451^#^
Radial artery occlusion	1 (1.8)	5 (9.6)	1.774	0.183^#^

# Fisher’s Exact Test. TRA: transradial aceess; dTRA: distal transradial aceess.

## DISCUSSION

This retrospective analysis demonstrated that dTRA offers certain advantages over conventional TRA in PCI for CHD, including shorter postoperative compression time and a lower overall incidence of complications.

The study found that although dTRA was associated with a significantly higher puncture time than TRA, the postoperative compression time was considerably lower in patients who underwent dTRA. This is consistent with the research results of Xiong et al.^11^ and Chen et al.^12^ The dTRA is relatively thinner and more superficial, making it slightly more challenging to locate the puncture point at the anatomical snuffbox region of the distal radial artery. This requires higher operational skills and experience from the attending physician, resulting in longer puncture time.^11^,^12^ Moreover, the superficial location of the dTRA facilitates easier compression and hemostasis following the procedure. In addition, the surrounding tissue in this region is relatively loose, which helps reduce patient discomfort during prolonged compression.^10^-^12^

The study found no significant differences between dTRA and TRA in terms of initial puncture success rate, puncture frequency, contrast agent dosage, X-ray exposure time, and surgical time. This indicates that the two access methods have similarities in the core aspects of operation and overall surgical efficiency. However, a study by Babunashvili et al.^13^ found that dTRA was associated with a significantly lower initial puncture success rate than TRA (70.9% vs. 83.9%). In this study, the initial success rates of dTRA and TRA were 87.3% and 92.6%, respectively, consistent with previous meta-analysis results.^14^,^15^ From an anatomical perspective, dTRA is slightly smaller and deeper than TRA, making catheterization potentially challenging.^14^-^16^ Although mastering dTRA requires a longer learning curve, this limitation can be addressed by promoting small vessel puncture under ultrasound guidance.

There was no significant difference in the incidence of single complications such as radial artery spasm, local hematoma, pseudoaneurysm, arteriovenous fistula, and RAO between dTRA and TRA. However, the total number of complications in dTRA was significantly lower than that in TRA, consistent with the research results of Zhuang et al.^17^ and Wang et al.^18^ From an anatomical perspective, the dTRA has a shallow surface and is located in an area with relatively fewer peripheral nerves, which reduces the probability of damage to surrounding tissues during puncture. Therefore, arterial spasm and local hematoma are significantly reduced.^17^,^18^ In the patients who underwent TRA in this study, the incidence of postoperative RAO was 9.6% (5/52). The main factors affecting RAO are the proximity of the puncture site to the wrist joint, complex surrounding structures, and prolonged postoperative compression leading to reduced blood flow.^17^-^20^ However, there was no statistically significant difference in the incidence of RAO between dTRA and TRA. This result is consistent with the DISCO RADAL study by Aminian et al.^20^ and further suggests that dTRA is safe in CHD patients undergoing PCI.

Based on the results of this study and previous research,^21^,^22^ the following nursing strategies may be proposed to prevent and reduce the occurrence of complications of dTRA or TRA:

### Preoperative:

A comprehensive assessment of the patient’s physical condition and underlying disease history; Using the Allen test and ultrasound to evaluate vascular conditions; Pay attention to psychological state, communicate and implement relaxation training to alleviate anxiety. At the same time, dietary guidance and reminders are given to patients to take antiplatelet and anticoagulant drugs on time.

### Intraoperative:

Assist in puncture positioning, improve success rate through touch and ultrasound; Familiar with the process and timely transfer of instruments to shorten the surgical duration; Closely monitor the patient’s vital signs to prevent the occurrence of cardiovascular complications.

### Postoperative:

Choose an appropriate compression hemostasis method; Pay attention to bleeding, hematoma, RAO, and other complications; Guide patients to adjust their physical activity, diet, and lifestyle. In summary, this study confirms that dTRA has the characteristics of “safe, efficient, and fast postoperative recovery” in coronary heart disease PCI. Especially in reducing postoperative compression time and complications, it has significant advantages, providing more optimized puncture path selection for clinical practice. Its value lies not only in the improvement of short-term efficacy, but also in promoting the development of interventional therapy towards “minimally invasive, precise, and comfortable” direction. It has important clinical practice guidance significance and promotion potential.

### Limitations:

First, it was conducted at a single center with a relatively small sample size (55 vs. 52), which may limit the generalizability of the findings. Patients who experienced access failure, underwent crossover, or had aborted PCI procedures were excluded, potentially introducing selection bias. The lack of subgroup analyses based on age, sex, or comorbidities such as diabetes was due to the limited number of complication events. Additionally, no sample size calculation or power analysis was performed, which may reduce the ability to detect subtle but clinically relevant differences. As a retrospective study, formal blinding during data analysis was not applied; however, analysts were independent of treatment and group allocation, and data were extracted from medical records to minimize observer bias. Finally, all procedures were performed by experienced operators, and no inter-operator variability analysis was conducted. Future prospective multicenter studies with larger samples are needed to validate these findings and explore operator-related effects and subgroup-specific outcomes.

## CONCLUSION

This study further confirms that dTRA has certain advantages in the PCI treatment of CHD, especially in terms of postoperative compression time and reducing the overall incidence of complications. However, due to the relatively small size of the dTRA blood vessels, more caution is needed when selecting instruments to ensure they match the blood vessels. Further high-quality research is required to refine the clinical application scenarios and timing of dTRA procedures.

### Future Recommendations:

Future studies are recommended to assess the safety and efficacy of dTRA in high-risk subgroups, such as elderly patients, those with diabetes, or advanced cardiac dysfunction. Long-term follow-up should also be conducted to evaluate delayed complications, particularly radial artery occlusion and vascular remodeling.

### Authors’ contributions:

**DG and GC:** Study design, Literature search and manuscript writing.

**GC, FL and QZ:** Data collection, data analysis and interpretation. Critical review

**DG:** Manuscript revision and validation and is responsible for the integrity of the study.

All authors have read and approved the final manuscript.
